# Supplementary material for: The integration of Gaussian noise by long-range amygdala inputs in frontal circuit promotes fear learning in mice
Source: eLife. 2020 Nov 30;9:e62594. doi: 10.7554/eLife.62594 (PMC7704104; doi:10.7554/eLife.62594)
Supplement: Transparent reporting form [file elife-62594-transrepform.docx]

***eLife’s* transparent reporting form**

We encourage authors to provide detailed information *within their submission* to facilitate the interpretation and replication of experiments. Authors can upload supporting documentation to indicate the use of appropriate reporting guidelines for health-related research (see [EQUATOR Network](http://www.equator-network.org/%20)), life science research (see the [BioSharing Information Resource](https://biosharing.org/)), or the [ARRIVE guidelines](http://www.plosbiology.org/article/info:doi/10.1371/journal.pbio.1000412) for reporting work involving animal research. Where applicable, authors should refer to any relevant reporting standards documents in this form.

If you have any questions, please consult our Journal Policies and/or contact us: [editorial@elifesciences.org](mailto:editorial@elifesciences.org).

**Sample-size estimation**

- You should state whether an appropriate sample size was computed when the study was being designed
- You should state the statistical method of sample size computation and any required assumptions
- If no explicit power analysis was used, you should describe how you decided what sample (replicate) size (number) to use

Please outline where this information can be found within the submission (e.g., sections or figure legends), or explain why this information doesn’t apply to your submission:

- No statistical methods were used to estimate sample size *a priori*, but β-power values were calculated (only for parametric tests).
- All Data (including the exact sample size for each experimental group/condition) and statistical tests (including description of any assumptions or corrections, such as tests of normality and adjustment for multiple comparisons, and a full description of the statistical parameters including central tendency (mean, median) and variation (standard deviation, sem) or associated estimates of uncertainty (interquartile range)) are provided in the figures and in the source tables.

**Replicates**

- You should report how often each experiment was performed
- You should include a definition of biological versus technical replication
- The data obtained should be provided and sufficient information should be provided to indicate the number of independent biological and/or technical replicates
- If you encountered any outliers, you should describe how these were handled
- Criteria for exclusion/inclusion of data should be clearly stated
- High-throughput sequence data should be uploaded before submission, with a private link for reviewers provided (these are available from both GEO and ArrayExpress)

Please outline where this information can be found within the submission (e.g., sections or figure legends), or explain why this information doesn’t apply to your submission:

- For each experiment, the number of replication is reflected by the number of recorded mice/cells used the study. Statistical tests were done on the whole population of cells/mice per experiments.
- No data were excluded from the analysis.

**Statistical reporting**

- Statistical analysis methods should be described and justified
- Raw data should be presented in figures whenever informative to do so (typically when N per group is less than 10)
- For each experiment, you should identify the statistical tests used, exact values of N, definitions of center, methods of multiple test correction, and dispersion and precision measures (e.g., mean, median, SD, SEM, confidence intervals; and, for the major substantive results, a measure of effect size (e.g., Pearson's r, Cohen's d)
- Report exact p-values wherever possible alongside the summary statistics and 95% confidence intervals. These should be reported for all key questions and not only when the p-value is less than 0.05.

Please outline where this information can be found within the submission (e.g., sections or figure legends), or explain why this information doesn’t apply to your submission:

- All data (including the exact sample size for each experimental group/condition) and statistical tests (including description of any assumptions or corrections, such as tests of normality and adjustment for multiple comparisons, and a full description of the statistical parameters including central tendency (mean, median) and variation (standard deviation, sem) or associated estimates of uncertainty (interquartile range)) are provided in the figures and in the supplemental table 1.
- Statistical procedures are provided in the method section: “Data are presented as the median ± interquartile range or mean ± sem (except where stated differently). All statistics were performed using Matlab (Mathworks) and Sigmaplot (Systat) with an α significant level set at 0.05. Normality of all value distributions and the equality of variance between different distributions were first assessed by the Shapiro-Wilk and Levene median tests, respectively. Standard parametric tests were only used when data passed the normality and equal variance tests. Non-parametric tests were used otherwise. Only two-sided tests were used. When applicable, pair-wise multiple post-hoc comparisons were done by using the Holm-Sidak method.”

(For large datasets, or papers with a very large number of statistical tests, you may upload a single table file with tests, Ns, etc., with reference to sections in the manuscript.)

**Group allocation**

- Indicate how samples were allocated into experimental groups (in the case of clinical studies, please specify allocation to treatment method); if randomization was used, please also state if restricted randomization was applied
- Indicate if masking was used during group allocation, data collection and/or data analysis

Please outline where this information can be found within the submission (e.g., sections or figure legends), or explain why this information doesn’t apply to your submission:

- Randomization methods were not used. Mice were allocated to experimental groups depending on their genotype (e.g. ArchT vs GFP) or learning phase (e.g. before vs after learning; naïve vs. conditioned).
- However, experiments and analysis were conducted blind to the operator.
- Statistical groups are indicated in the figures and in the source tables.

**Additional data files (“source data”)**

- We encourage you to upload relevant additional data files, such as numerical data that are represented as a graph in a figure, or as a summary table
- Where provided, these should be in the most useful format, and they can be uploaded as “Source data” files linked to a main figure or table
- Include model definition files including the full list of parameters used
- Include code used for data analysis (e.g., R, MatLab)
- Avoid stating that data files are “available upon request”

Please indicate the figures or tables for which source data files have been provided:

All numerical data are provided in the source tables
